# A Meta-Analysis of Enteral Nutrition and Total Parenteral Nutrition in Patients with Acute Pancreatitis

**DOI:** 10.1155/2011/698248

**Published:** 2011-06-02

**Authors:** Heming Quan, Xingpeng Wang, Chuanyong Guo

**Affiliations:** Department of Gastroenterology, Tenth People's Hospital, Tongji University, Shanghai 200072, China

## Abstract

*Objective*. To analyze the effect of total parenteral nutrition (TPN) and enteral nutrition (EN) in patients with acute pancreatitis. *Methods*. Randomized controlled trials of TPN and EN in patients with acute pancreatitis were searched in NCBI and CBM databases and The Cochrane Controlled Trials Register. Six studies were enrolled into the analysis, and the details about the trial designs, characters of the subjects, results of the studies were reviewed by two independent authors and analyzed by STATA 11.0 software. *Results*. Compared with TPN, EN was associated with a significantly lower incidence of pancreatic infection complications (RR = 0.556, 95% CI 0.436*∼*0.709, *P* = .000), MOF (RR = 0.395, 95% CI 0.272*∼*0.573, *P* = .003), surgical interventions (RR = 0.556, 95% CI 0.436*∼*0.709, *P* = .000), and mortality (RR = 0.426, 95% CI 0.238*∼*0.764, *P* = .167). There was no statistic significance in non-pancreatitis-related complications (RR = 0.853, 95% CI 0.490*∼*1.483, *P* = .017). However, EN had a significantly higher incidence of non-infection-related complications (RR = 2.697, 95% CI 1.947*∼*3.735, *P* = .994). *Conclusion*. EN could be the preferred nutrition feeding method in patients with acute pancreatitis.

## 1. Introduction

Acute pancreatitis is an inflammatory process that presents different severity degrees, ranging from a mild self-limited disease, with interstitial edema in the pancreas, to a severe disease with extensive necrosis [1]. Severe AP with its related systemic inflammatory response (SIR) causes increased metabolic demands and may progress to multiorgan disease (MOD). Cigarette smoking is an independent risk factor for AP (95% confidence interval 1.48 to 3.09) and total exposure correlates with overall risk [2]. Recurrent attacks of alcoholic AP, however, were not associated with cigarette smoking [3] but occurred less frequently in those who had repeated 6-month counselling sessions (to encourage sobriety) compared with a single session (8% versus 21%, *P* = .042) [4]. These data reinforce the common sense approach of encouraging drinking cessation.

The clinical course of an attack of AP varies from a short period of hospitalization with supportive care to prolonged hospitalization and admittance to an intensive care unit (ICU) because of the development of systemic inflammatory response syndrome (SIRS), multiorgan failure (MOF), and septic complications. Overall, in about 15% to 20% of patients, AP progresses to a severe illness with a prolonged disease course. These severely ill patients may develop organ failure and/or local complications such as pancreatic necrosis. Approximately 75% of the patients have mild disease with mortality below 1% [5]. Mortality increases up to 20% if the disease progresses to its severe necrotizing form [6–11], and in the most severe cases mortality can range from 30 to 40% [12, 13]. In severe necrotising pancreatitis, 80% of all patients are catabolic, with high energy expenditure and enhanced protein catabolism. The negative nitrogen balance can be as much as 40 g/day and can have a deleterious effect on both nutritional status and disease progression [14–16]. The traditional treatment strategy is total parenteral nutrition (TPN) giving the inflamed pancreas a rest. Thus, nutritional supply is easily controlled, and adynamic ileus and pancreatic stimulation are avoided. However, in addition to cost and the risk of catheter-related sepsis, TPN may worsen the inflammatory process, lead to metabolic and electrolyte disturbances, alter gut barrier due to increased intestinal permeability, and develop sepsis and multiple organ failure [17]. 

Many high-quality studies have demonstrated that EN results in clinically relevant and statistically significant risk reduction of infectious complications, pancreatic infections, and mortality in patients with predicted severe acute pancreatitis. EN has also been shown to be beneficial as an adjunct to the management of severe acute pancreatitis by obviating the systemic inflammatory response syndrome and in modifying the course of the disease. On the other hand, TPN has a deleterious effect on the intestinal barrier function. Studies performed on animals and human beings show that TPN may worsen intestinal atrophy and intestinal and systemic immunity, and thereby it may also contribute to impair the intestinal mucosa permeability and facilitate bacterial translocation [1]. The protective role of EN, compared with parenteral nutritional (PN), in maintaining the integrity of the gut barrier has been demonstrated in a rat model of acute pancreatitis. The EN group was found to have significantly less bacterial translocation and a lower blood endotoxin level than the PN group.

Data of randomized controlled trials have compared the effect of TEN and TPN. But the results are always not the same and the effect of therapeutics is still not identified because of the small sample sizes. Here, we conducted meta-analysis comparing enteral nutrition with parenteral nutrition to determine the incidence of complications associated with these different routes of nutritional support [18].

## 2. Methods and Materials

### 2.1. Inclusion Criteria

All clinical randomized controlled trials are performed on adults with predicted severe acute pancreatitis and reported in English. In each case, the definition of the complication was taken as that given in the primary trial. The safety outcome included at least one of the following: pancreatitis-related complications, non-pancreatitis-related complications, non-infection-related complications, multiple-organ failure (MOF), surgery intervention, hospital stay, and mortality.

### 2.2. Exclusion Criteria

The comparison not between TPN and EN, such as the comparison in TPN, EN and other traditional trials. The outcome included none of the above.

### 2.3. Material Collection

We collected all the studies of randomized controlled trials that compared the effect of TEN and TPN. Data from full-text articles were retrieved and checked for consistency by two of us (Heming Quan and Chuanyong Guo) independently. The NCBI (1966–2010) and CBM (2005–2010) databases and The Cochrane Controlled Trials Register [19] were cross-searched for articles. A bibliographic search in the databases was made using the following predefined terms: (acute [All Fields] AND severe [All Fields] AND (“pancreatitis” [MeSH Terms] OR “pancreatitis” [All Fields]) AND (“enteral nutrition” [MeSH Terms] OR (“enteral” [All Fields] AND “nutrition” [All Fields]) OR “enteral nutrition” [All Fields])) OR ((“jejunum” [MeSH Terms] OR “jejunum” [All Fields] OR “jejunum” [All Fields]) AND (“enteral nutrition” [MeSH Terms] OR (“enteral” [All Fields] AND “nutrition” [All Fields]) OR “enteral nutrition” [All Fields] OR (“tube” [All Fields] AND “feeding” [All Fields]) OR “tube feeding”[All Fields])) OR (nasogastric [All Fields] AND (“enteral nutrition” [MeSH Terms] OR (“enteral” [All Fields] AND “nutrition” [All Fields]) OR “enteral nutrition” [All Fields] OR (“tube” [All Fields] AND “feeding” [All Fields]) OR “tube feeding” [All Fields])) AND (“humans” [MeSH Terms] AND Clinical Trial [ptyp] AND English [lang] AND “2000/01/1” [PDat] : “2010/12/31” [PDat]). Six randomized controlled trials from six countries were included in our study.

### 2.4. Quality Evaluation

The methodologic quality of the studies was assessed using a previously published scoring system, with a quality scale range of 0 to 5 points according to Jadad score system [16, 20]. This quality scale incorporates method of randomization, masking, and dropouts and withdrawals. A score of 2 or less represents a low-quality study, whereas a score of at least 3 represents a high-quality study. The results of Jadad score are presented in Table 2.

### 2.5. Data Collection

The following parameters were extracted: design of trials, population, incidence of pancreatitis, way of nutrition support, and the outcome of these criteria. We calculated the incidence of pancreatitis according to APACHE II score, the level of CRP, and the incidence of CT. The population statistics results included in the study are presented in Tables 1(a) and 1(b). The effective endpoints of every trial are presented in Table 3.

### 2.6. Data Analysis

We analyzed pancreatitis-related complications, non-pancreatitis-related complications, non-infection-related complications, surgery intervention, multiple-organ failure (MOF), and mortality between TEN and TPN. The data analysis and graphs were performed using the M-H model with STATA 11.0 (Stata Corp., College Station, Tex, USA). We tested heterogeneity between trials with *χ* ^2^ tests, with *P* ≤ .01 indicating significant heterogeneity. The relative risk (RR) was presented with 95% confidence interval (CI) for dichotomous data using M-H model. Besides, hospital stay was analyzed as a continuous variable.

## 3. Results

**Figure 1:**
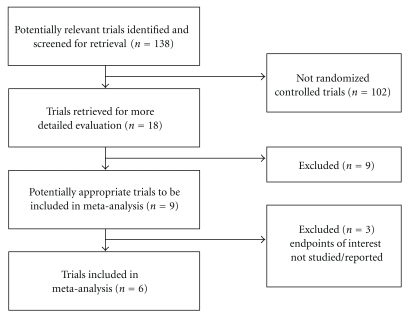
Process of study selection of RCTs.

Of 138 articles screened, 9 RCTs comparing EN and TPN were identified. Only 6 RCTs [20, 22–26] fulfilled the criteria in the meta-analysis. 6 RCTs were included because: (1) an Acute Physiology and Chronic Health Evaluation (APACHE II) score ≥ 8; (2) trial group received TEN and control group received TPN; (3) effective ends include one of pancreatitis-related complications, non-pancreatitis-related complications, non-infection-related complications, surgery intervention, multiple-organ failure (MOF), and mortality. Five of the six studies are high-quality studies [21]. Random generation: two studies were opaque envelopes, one study was odd/even numbers, and two studies were computerized random number generation. Blind: all patients were randomized to receive either enteral or parenteral feeding, so none of the studies was blind. Baseline characteristics: all of the six studies reported the baseline characteristics of the two groups, four of which performed statistics analysis. The results indicated that the baseline demographic and clinical characteristics of the two groups were similar. No significant differences were found between the two groups of patients. Loss of followup: all patients in the six studies were hospitalized and followed up during the study. No patients were failed to be followed up. Intention-to-treat analysis and compliance analysis are almost coincident. These characteristics are presented in Figure 1.

### 3.1. Pancreatitis-Related Complications

**Figure 2:**
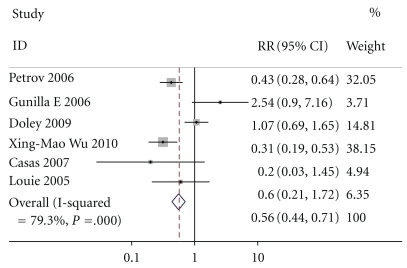


All studies reported pancreatitis-related complications, including pancreatic infection, pancreatic abscess, and pancreatic necrosis, not including infections out of pancreas. By M-H analysis, there was a significantly lower risk of infection in patients who received enteral nutrition compared to those who received parenteral nutrition (relative risk of 0.556, 95% confidence interval 0.436 to 0.709, *P* = .000, Figure 2). The test result for heterogeneity between the studies was not significant (*P* = .13006252). 

### 3.2. Non-Pancreatitis-Related Complications

**Figure 3:**
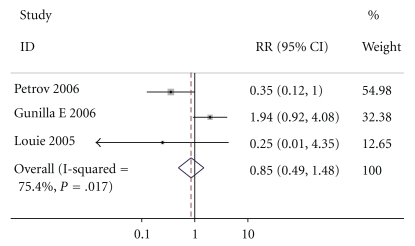


All studies reported non-pancreatitis-related complications, including pneumonia, urinary system infection, and central venous catheter infection. By M-H analysis, there was no statistic significance in non-pancreatitis-related complications in patients who received enteral nutrition compared to those who received parenteral nutrition (relative risk of 0.853, 95% confidence interval 0.490 to 1.483, *P* = .017, Figure 3). The test result for heterogeneity between the studies was not significant (*P* = .826279278). 

### 3.3. Non-Infection-Related Complications

**Figure 4:**
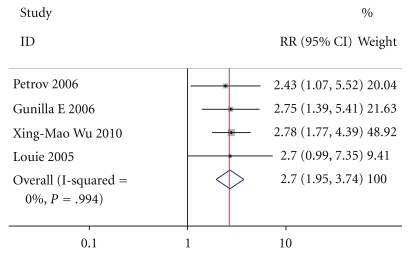


Except for Casas and Doley, four of the six studies intimately reported non-infection-related complications, including adult respiratory distress syndrome, pancreatic cyst and fistula, diarrhea, abdominal bloating, and remove of nasal jejunal tube, not including MOF By M-H analysis, there was a significantly higher risk in patients who received enteral nutrition compared to those who received parenteral nutrition (relative risk of 2.697, 95% confidence interval 1.947 to 3.735, *P* = .994, Figure 4). The test result for heterogeneity between the studies was significant (*P* = .008212507).

### 3.4. MOF

**Figure 5:**
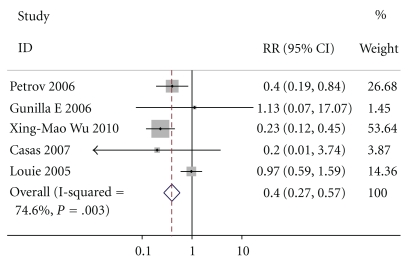


Except for Doley, five of the six studies intimately reported MOF, By M-H analysis, there was a significantly lower risk of mortality in patients who received enteral nutrition compared to those who received parenteral nutrition (relative risk of 0.395, 95% confidence interval 0.272 to 0.573, *P* = .003, Figure 5). The test result for heterogeneity between the studies was not significant (*P* = .307088503).

### 3.5. Surgical Interventions

**Figure 6:**
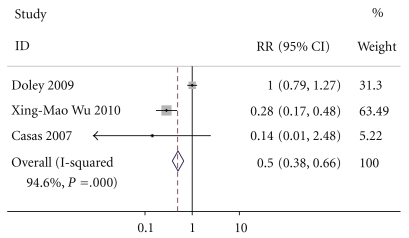


Except for Casas, Doley, and Wu, three of the six studies intimately reported surgical interventions. By M-H analysis, there was a significantly lower risk of surgical interventions in patients who received enteral nutrition compared to those who received parenteral nutrition (relative risk of 0.501, 95% confidence interval 0.378 to 0.663, *P* = .000, Figure 6). The test result for heterogeneity between the studies was not significant (*P* = .440677966).

### 3.6. Mortality

**Figure 7:**
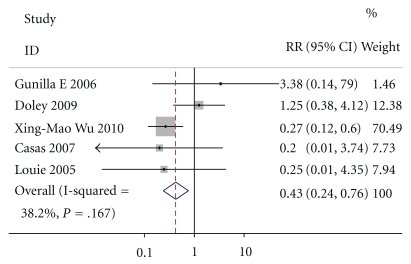


Except for Petrov, five of the six studies intimately reported mortality. By M-H analysis, there was a significantly lower risk of mortality in patients who received enteral nutrition compared to those who received parenteral nutrition (relative risk of 0.426, 95% confidence interval 0.238 to 0.764, *P* = .167, Figure 7). The test result for heterogeneity between the studies was not significant (*P* = .190278767).

### 3.7. Hospital Stay

Three of the six studies intimately reported hospital stay. This index is continuous variable. The two groups could not be compared because most of the studies did not provide intimate standard deviation. But according to the average hospital stay, there was no significant difference in hospital stay between the two groups. This result was also found in other studies [27, 28].

### 3.8. EN Regimen and Time

Except for Doley and Casas, the other papers all used semielemental nutrition as EN regimen. Most of them infused the nutrition at 25 mL/h and increased by 10 mL/h every 6 hours. Three of them initiate EN within 24 hours. EN was given for more than seven days in all the six papers (Table 4).

## 4. Discussion

This meta-analysis shows that EN, compared with PN, has important beneficial effects in patients with predicted severe acute pancreatitis, notably, clinically relevant, and statistically significant risk reduction in pancreatitis-related complications, non-pancreatitis-related complications, multiple-organ failure (MOF), surgery intervention, and mortality. But PN is superior to EN in non-infection-related complications. There is no difference between EN and PN in hospital stay. The literatures included in this meta-analysis, except for Wu, are all high-quality randomized controlled trials with Jadad score more than three and summarizing the best available evidence-based data. The statistic characterizations of the population and the inclusion/exclusion criteria of severe acute pancreatitis patients are also the same: (1) abdominal pain, (2) pancreatic enzymes three times higher than normal, and (3) APACHE II score ≥ 8. Patients in the two groups have no difference in age and gender. Their diseases are mostly caused by cholelithiasis and alcohol pancreatitis. This meta-analysis contains seven outcomes of effectiveness.

### 4.1. Pancreatitis-Related Complications and Non-Pancreatitis-Related Complications

To date, there is a substantial scientific evidence that enteral feeding is superior to total parenteral nutrition (TPN) [29, 30]. The beneficial effects of enteral feeding on mucosal integrity and the prevention of bacterial overgrowth may well explain the superiority of enteral feeding over TPN. Enteral feeding significantly reduces the risk of infections, lowers the need for surgical interventions, and reduces the length of hospital stay. In the past few years, it has been proposed that EN through nasogastric (NG) tubes may be a simple, safe, and equally valid alternative to nasojejunal tubes, with the potential advantage of earlier administration of nutrients. However, NG feeding cannot be recommended at this time, and it is not clear if a subgroup of SAP patients may benefit more from this approach [31–35].

The facts that EN is most likely superior to parenteral nutrition in preventing septic complications of acute pancreatitis, may also eliminate some complications of PN (catheter sepsis, pneumothorax, and thrombosis), and costs only 15% of the cost of TPN, make it an increasingly accepted treatment modality [36]. Windsor et al. randomized 34 patients to TPN or enteral nutrition for 7 days. They reported that the reduction in inflammatory response with enteral nutrition could be ascribed to the suppression of bacterial overgrowth rather than to the reduction in pancreatic injury. This observation was supported by the finding of (1) no increase in screen endotoxin antibodies in the enteral nutrition group as compared to an increase in the TPN group (*P* < .05) and (2) no difference in CT evaluation of the pancreatic injury after enteral nutrition or TPN. There is accumulating clinical evidence that enteral nutrition can improve survival and reduce the complications accompanying the severe acute pancreatitis. The explanations are complex and related to the fact that (1) enteral nutrition avoids TPN complications, (2) luminal nutrition maintains intestinal health, (3) enteral amino acids are more effective in supporting splanchnic protein synthesis, and (4) enteral nutrition may prevent the progression of multiple organ failure.

Many clinical and experimental studies have demonstrated that TPN can promote more cytokine production compared to EN [37, 38], including IL-6, IL-8, TNF, and CRP. Total parenteral nutrition has failed to show any clinical benefits for the patients, as it cannot protect the gut mucosa. On the contrary, enteral feeding repairs the mucosal damage of fasting. If enteral feeding is given very early, it may preserve epithelial integrity and bacterial ecology, thereby helping to maintain gut barrier function [39]. Doley et al. reported that patients given TPN were more often infected by Gram-positive organisms and fungi as compared to those given EN who were more often infected by a Gram-negative organism. This observation has an important bearing on the outcome of severe acute pancreatitis as fungal infection carries a higher risk of mortality. Though proper aseptic precautions were maintained, the occurrence of infections with Gram-positive organisms such as *Staphylococci* in the TPN group suggests that it could be due to the invasion of central line catchers with cutaneous commensals [21].

Petrov et al. reported that extrapancreatic infectious complications were more frequent in their study (22%) than those in other studies. In addition to the severity of the patients included, this is likely due to the prolonged hospital stay and maintained central venous and urinary catheters. In fact, catheter infection was the most frequent extrapancreatic infectious complication. In their study, 46% of patients were found to have infections, and the most common organisms were *Escherichia coli* and *Pseudomonas aeruginosa*, which is similar to earlier reports. It gives an idea about the possible source of infection. In their series, as well as others, most of the infections were with Gram-negative bacteria, and up to one-third of infections in their study were polymicrobial. This would suggest that the gut was the most likely source of infection [17].

### 4.2. Non-Infection-Related Complications

Compared to TPN, TEN has higher risk of non-infection-related complications caused by nutrition support. Although TEN has so many complications, such as diarrhea and abdominal distension, the risk is much lower than septicemia induced by tube. Recently, Karakan et al. reported that beneficial bacteria can reduce diarrhea complications induced by TEN [40]. Anyway, a number of these complications such as tube removal or diarrhea are relatively minor and can often be managed by avoiding liquid and elixir medications that contain sorbitol (for diarrhea) and use of a nasal bridle in patients at risk for tube removal.

### 4.3. MOF and Mortality

This meta-analysis demonstrates that TEN is superior to TPN in MOF and mortality. But Doley's study got the conclusion that there is no difference in mortality. But totally, we still support the conclusion that TEN is superior to TPN.

### 4.4. Surgical Interventions

The references included in our study all reported that TEN has less surgical interventions compared to TPN. But Doley's study got the conclusion that there is no difference in surgical interventions. This conclusion may be caused by the severity of the patient's condition.

## 5. Disadvantages

We did not perform sensitivity analysis because there is only on paper with the Jadad score less than two. But there are still many disadvantages in this meta-analysis. First, the population included in the study is small. Second, in Gunilla's study, the nutritional support way was nasogastric feeding tube. One new meta-analysis compared nasogastric feeding tube and nasojejunal feeding tube and showed that there was no difference between the two ways in mortality, hospital stay, infection-related complications, and costs [41]. Third, the total number of patients enrolled was limited (*n* = 335), which may lead to wide CI. Fourth, it is possible that studies with negative results, which showed no trend in favor of either intervention, may remain unpublished, leading to publication bias.

Taken together, this meta-analysis confirmed previous reports in the literature, especially those from recent years. This study demonstrated that enteral nutritional support is safe and effective when compared to parenteral support. Compared with TPN, EN was associated with a significantly lower incidence of pancreatic infection complications (RR = 0.556, 95% CI 0.436~0.709, *P* = .000), MOF (RR = 0.395, 95% CI 0.272~0.573, *P* = .003), surgical interventions (RR = 0.556, 95% CI 0.436~0.709, *P* = .000), and mortality (RR = 0.426, 95% CI 0.238~0.764, *P* = .167). There was no statistic significance in non-pancreatitis-related complications (RR = 0.853, 95% CI 0.490~1.483, *P* = .017). However, EN had a significantly higher incidence of non-infection-related complications (RR = 2.697, 95% CI 1.947~3.735, *P* = .994). There was no significant difference in hospital stay between patients with TPN and EN. However, a deficiency of this study is its retrospective nature and relatively small sample size. Therefore, studies with larger sample sizes should be done to better define the role of enteral nutritional support in the treatment of severe acute pancreatitis.

## Figures and Tables

**Table:** (a) Study characteristics of the included trials

Authors	Years	Country	No. Patients (EN/PN)	APACHE II score (EN/PN)	Age (EN/PN)	Gender (EN/PN)	
						Male	Female
Louie [23]	2005	UK	10/18	11.8/12.7	65.3/59.0	6/9	4/9
Petrov [25]	2006	Russia	35/34	12/12.5	51/52	27/24	8/10
Gunilla [24]	2006	Sweden	24/26	10/9	71/68	10/14	14/12
Casas [26]	2007	Spain	11/11	>8	61.2/55.6	8/8	3/3
Doley [20]	2009	India	25/25	>8	38.4/41.1	—	—
Wu [22]	2010	China	53/54	14/16	52/54	32/21	30/24

**Table:** (b) Study characteristics of the included trials

Authors	Years	Biliary etiology (EN/PN)			Ways of nutritional support
		Cholelithiasis	Alcohol	Others	

Louie	2005	5/7	2/4	3/7	Nasojejunal feeding tube, endoscopy
Petrov	2006	11/13	16/15	8/6	Nasojejunal feeding tube, X-ray
Gunilla	2006	14/17	3/4	7/5	Nasogastric feeding tube
Casas	2007	4/7	1/4	6/0	Nasojejunal feeding tube, endoscopy
Doley	2009	10/13	11/8	4/4	Nasojejunal feeding tube, endoscopy
Wu	2010	—	—	—	Nasojejunal feeding tube, endoscopy

**Table 2:** Jadad score.

Authors	Years	Randomized method	Blind	Exit/lost to followup	Jadad scores
Louie	2005	3-center, computer-generated assignment placed in sealed, opaque envelopes	No	Yes	3
Petrov	2006	Computerized random number generation	No	Yes	3
Gunilla	2006	Single-center, sealed, numbered envelopes	No	Yes	3
Casas	2007	Computerized random number generation	No	Yes	3
Doley	2009	Odd/even numbers	No	Yes	3
Wu	2010	Not mentioned	No	Yes	2

**Table:** (a)

Authors	Years	No. Patients (EN/PN)	Pancreatitis-related complications (EN/PN)	Non-pancreatitis-related complications (EN/PN)	Non-infection-related complications (EN/PN)
Louie	2005	10/18	3/9	0/3	6/4
Petrov	2006	35/34	14/32	4/11	15/6
Gunilla	2006	24/26	9/4	12/7	17/7
Casas	2007	11/11	1/5	—	—
Doley	2009	25/25	16/15	—	—
Wu	2010	53/54	12/39	—	41/15

**Table:** (b)

Authors	Years	MOF (EN/PN)	Mortality (EN/PN)	Hospital stay (EN/PN)	Surgery intervention (EN/PN)
Louie	2005	7/13	0/3	—	—
Petrov	2006	7/17	—	—	—
Gunilla	2006	1/1	1/0	—	—
Casas	2007	0/2	0/2	30.2/30.7	0/3
Doley	2009	—	5/4	42/36	21/21
Wu	2010	8/35	6/23	27/16	12/43

**Table 4:** EN regimen and time.

Author	Year	EN regimen	Initial time	Lasting time
Louie	2005	A semielemental product with low fat content was infused at 25 mL/h and increased by 10 mL/h every 6 hours, until the target rate was achieved	Within 24 h	10 days

Petrov	2006	Semielemental nutrition was commenced at a rate of 25 mL/h and increased by 10 mL/h every 6 h, until the desired caloric intake was reached	Within 48 h	More than 7 days

Gunilla	2006	Semielemental nutrition rate was 25 mL/hr and gradually increased daily up to 100 mL/hr if tolerated and needed. The aim was to reach full nutrition within 72 hours	Within 24 h	10 days

Casas	2007	Polymeric diet infusion rate was 25 mL/h with increases of 25 mL/4 h until requirements were reached	Within 72 h	More than 10 days

Doley	2009	Not mentioned	Within 72 h	More than 14 days

Wu	2010	Semielemental nutrition was given at 20 mL/h for 20 hours	Within 24 h	16 days
